# Behavioral and Mental Wellbeing in Indigenous Communities: A Protocol for Developing an Educational Program for Nurses, Community and the Health Care Workforce Serving Indigenous Communities

**DOI:** 10.3390/nursrep16080260

**Published:** 2026-07-28

**Authors:** Michelle Kahn-John, Brinda Sivaramakrishnan, Katie E. Nelson, Joshuaa Allison-Burbank, Nora Bambrick, Rita D’Aoust, Lisa Dickson, Mina Kazemi, Deserae Kill Eagle, Karan Kverno, Jessica Meese, Julie Nanavati, Tamar Rodney, Janai Williams, Teresa Brockie

**Affiliations:** 1 Johns Hopkins School of Nursing, Baltimore, MD 21205-2110, USA; knelso46@jhmi.edu (K.E.N.); hbambri1@jh.edu (N.B.); rdaoust1@jhu.edu (R.D.); ldickso8@jh.edu (L.D.); mkazemi2@jhmi.edu (M.K.); dkillea1@jh.edu (D.K.E.); kkverno1@jh.edu (K.K.); jenanavati@jhu.edu (J.N.); trodney1@jhu.edu (T.R.); janaiwilliams19@gmail.com (J.W.); tbrocki1@jhu.edu (T.B.); 2 Center for Indigenous Health, Johns Hopkins University Bloomberg School of Public Health, Baltimore, MD 21210-9997, USA; bsivara2@jh.edu (B.S.); jalliso8@jhu.edu (J.A.-B.); jmeese1@jh.edu (J.M.)

**Keywords:** Indigenous mental health, American Indian health, culturally safe care, workforce development, task shifting, mental health literacy, suicide prevention, curriculum adaptation, community-led training, care coordination

## Abstract

**Background/Objective:** The Behavioral and Mental Wellness in Indigenous Communities (BMWIC) education program was created to address mental health (MH) resource gaps in the Fort Belknap Community (FBC). Designed to enhance MH literacy, task-shifting capacity, and MH care coordination skills, the FBC partnered with Johns Hopkins School of Nursing (JHSON) to co-develop a culturally aligned MH training program. This protocol outlines the development of the BMWIC curriculum. **Methods:** Steps 1–5 of the 6-step Collaborative Participatory Adaptation Model (CPAM) guided the development of the BMWIC, including collaboration, review of evidence-based models, cultural adaptation of materials, and the creation of a robust evaluation plan to be conducted. Four courses were ultimately developed on: historical trauma, culturally informed MH screening and care, risk and protective factors and health-related Tribal, state and federal legal jurisdiction in American Indian and Alaska Native (AI/AN) communities. **Discussion:** The BMWIC development process serves as a prime example of translating community priorities into an educational intervention through a combination of best practices in health science education with Indigenous ways of knowing (IWK). Targeted training and task shifting, such as through the BMWIC, may have the potential to expand MH-related knowledge and strengthen the capacity of community health systems to identify psychological and behavioral concerns early, coordinate care more effectively, and improve access to MH care and resources at the community level. **Conclusions:** This protocol may be useful for academic-community partnerships creating culturally informed educational programs across disciplines and specialties in service to Indigenous communities. The next steps will involve piloting and evaluating the curriculum and collaborating with healthcare organizations to determine sustainable pathways for scaling the BMWIC where it is most needed.

## 1. Introduction

Mental health (MH) resource gaps are prevalent in rural, remote, low-income American Indian and Alaska Native (AI/AN) communities. At least 40% of the U.S. population resides in a Mental Health Professional Shortage area (MH HPSA) and the shortage of MH professionals is greater in rural and remote communities [1]. Other factors that are widening the MH resource gap in AI/AN communities include the scarcity of culturally aligned programs, low MH literacy (community wide and among healthcare workers), and the shortage of qualified AI/AN and/or culturally informed MH professionals. An additional concern is caregiver burnout experienced by healthcare providers working in AI/AN communities [2].

Per treaty obligations originating in 1851, the United States Government provides healthcare services for AI/AN communities. Despite existing healthcare services, the impact of colonization and historical trauma has been detrimental to the health and well-being of AI/AN communities. Socioecological determinants contributing to inequitable health outcomes include poverty, housing instability, food insecurity, limited access to care and education, discrimination, and disrupted cultural and social support systems [3]. Elevated rates of substance use, posttraumatic stress, interpersonal violence, and suicide are well documented as MH disparities affecting AI/AN populations [4,5]. National data show striking inequities in suicide mortality and age-adjusted suicide rates among AI/AN people, which are substantially higher than those for other racial and ethnic groups. Suicide mortality among AI/ANs is 3.5 times higher than the general population [4,5]. Youth suicide is significant among these disparities and is the second leading cause of death for AI/AN people ages 10 to 34 [4,5].

MH and nursing positions remain difficult to fill in rural and remote underserved communities, thereby compelling approaches taken toward enhancing education and task shifting [1,6]. Increasing clinical MH literacy among community members, healthcare workers and front-line responders (i.e., CHWs, nurses) may address existing MH resource gaps in AI/AN communities [7,8]. In a study conducted by Brockie et al., participants confirmed severe MH workforce shortages exist in the Fort Belknap Community [9].

Further complicating the gaps in MH literacy among healthcare professionals is the need for education on diverse health systems and cultural safety [10]. Though academic institutions have recently started to include cultural safety content, health science education has historically excluded topics on Indigenous ways of knowing (IWK) and prioritized dominant educational frameworks [11,12]. The exclusion of diverse knowledge systems creates a healthcare workforce that is uninformed on the tenets and necessity of culturally safe healthcare. Dr. Irihapeti Ramsdem, a Māori nurse from Aotearoa in New Zealand, introduced the concept of cultural safety in education and nursing practice. She states: “Culturally safe teaching and clinical practice require a level of self-reflection by the educator or health care provider to identify one’s biases and attune styles of teaching and clinical practice that respectfully aligns with the person or population being served” [13]. The capacity of nurses, caregivers, and the healthcare workforce to self-reflect, identify biases, and attune care to the culturally diverse needs of those they serve is an ever-evolving skill. Despite slight international progress in developing culturally safe educational environments, systemic and anti-Indigenous racism persists [14,15,16]. This is evident within education systems which can constrain or block access to culturally safe educational opportunities [10].

**Figure 1:**
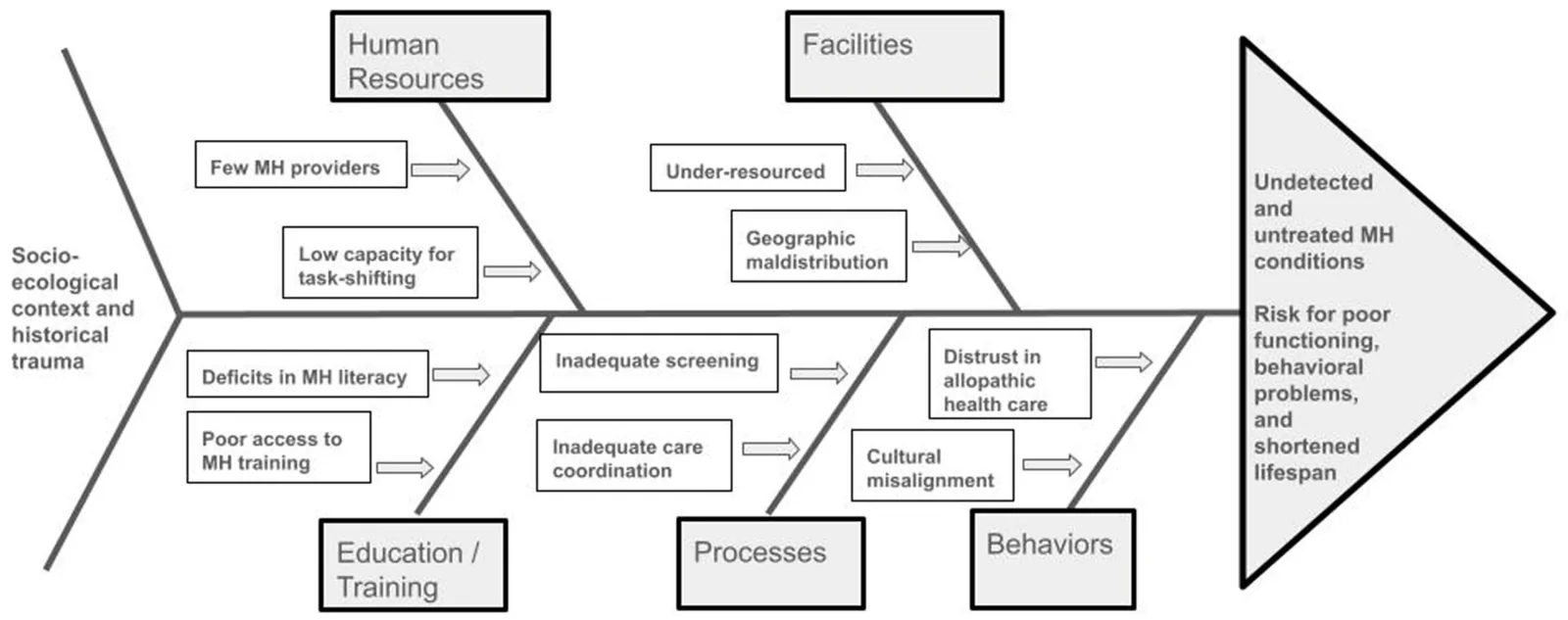
Barriers to accessing mental health resources. Shaded boxes in the “bones” represent contributing mental health barrier categories, the white boxes represent specific barriers, and the shaded “head” represents existing MH challenges and disparities.

Figure 1 depicts the social, environmental, and workforce barriers to MH resources and access to MH care reported by AI/AN communities. The fishbone diagram illustrates how MH resource gaps in AI/AN communities stem from broader socioecological realities and historical trauma. The “bones” identify key contributing areas, including human resources, facilities, education/training, processes, and behaviors. The socioecological realities in AI/AN communities contribute to having few MH providers, under-resourced and unevenly distributed facilities, limited access to training, inadequate screening and care coordination, and distrust or cultural misalignment in care. Together, these factors compound the risks of undetected and untreated MH conditions.

Existing evidence indicates that MH resource gaps can be addressed through systems-level strategies such as task shifting and community-based education programs [17]. Task shifting is the intentional and supervised transfer of health care duties from more to less specialized workers (including laypersons) [17]. For example, rather than having to rely on a specialist, a CHW may facilitate MH-related tasks within their role scope. Nurses and CHWs in AI/AN communities are uniquely suited to extend clinical reach across the MH resource gap via relational and cultural ties [18,19,20]. However, transferring duties requires increasing knowledge and skills among those without MH training, so they may learn to effectively identify and address MH-related needs from both cultural and clinical perspectives. This approach fortifies the infrastructure of under-resourced health systems by increasing access to MH resources while expanding care coordination capacity for existing healthcare providers [21].

Populations with complex MH risk factors need immediate access to services, as well as relevant MH training for nurses, CHWs, healthcare providers, community members, and other essential first responders. Innovative strategies are needed to close the MH resource gap, including the strategic use of available human resources within AI/AN communities. Culturally aligned training opportunities focused on MH promotion, early identification of psychological and behavioral symptoms, the use of screening tools and MH care coordination may be beneficial for communities experiencing significant MH challenges.

### Partner Engagement: Fort Belknap Community and Johns Hopkins School of Nursing

In 2019, the Fort Belknap community (FBC) in Northern Montana experienced a youth suicide cluster. Few MH providers were available to address the MH needs of a population of more than 6000 people. FBC nurses and CHWs are highly valued and trusted members of the community who have a wide range of interpersonal and clinical skills, understand the culture, and are valuable resources when services and specialists are scarce. The FBC identified gaps in knowledge about promoting mental health and recognizing early signs of psychological and behavioral distress among frontline healthcare workers. Leaders were also concerned about heavy workloads and caregiver burnout among FBC nurses. In response to the mental health needs of the community, including nurses, CHWs and other healthcare workers, Fort Belknap Tribal Leadership partnered with the Johns Hopkins School of Nursing (JHSON) to address these resource gaps.

A core component of this collaborative partnership is described in this protocol: the development of a tailored educational program to increase MH knowledge and skills of FBC nurses, CHWs, and community members working with the Aaniiih and Nakoda Tribes.

## 2. Materials and Methods

### 2.1. Collaborative Participation Adaptation Model (CPAM)

**Figure 2:**
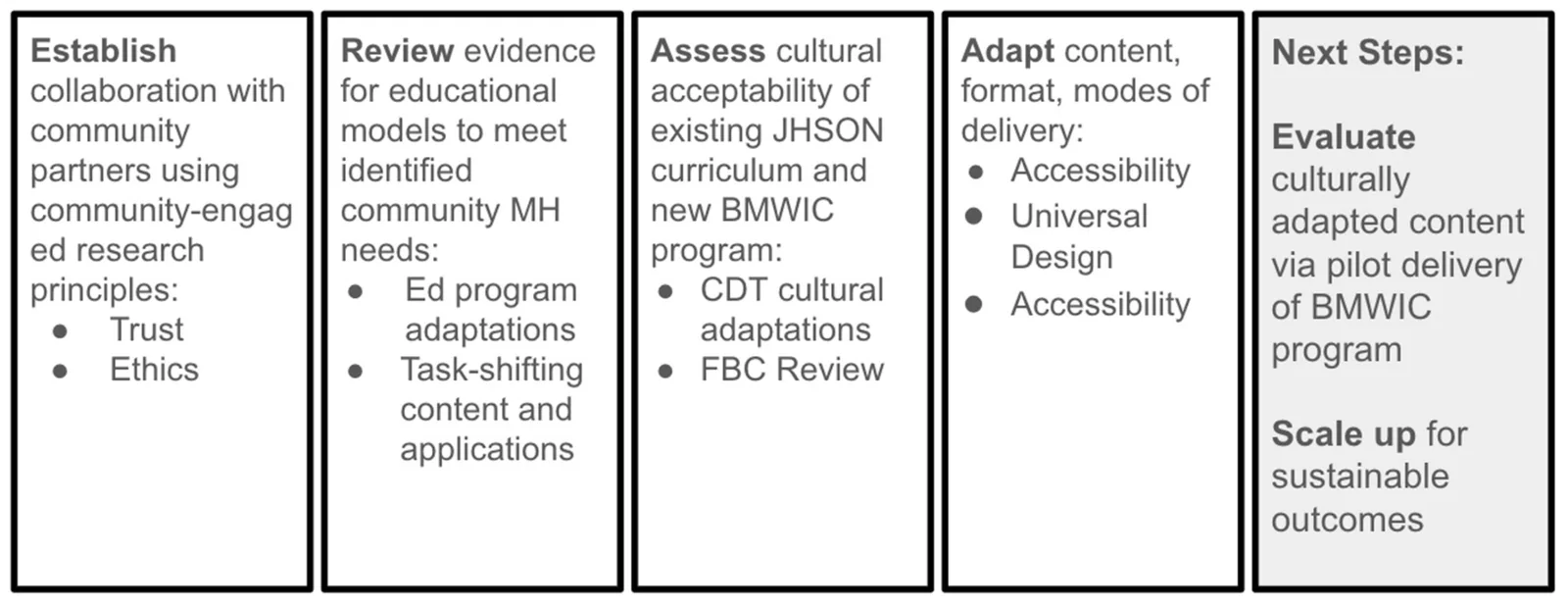
CPAM stepwise process. White boxes indicate completed steps; grey indicates those still to come.

The Behavioral/Mental Wellness in Indigenous Communities (BMWIC) program team utilized the Collaborative Participation Adaptation Model (CPAM) as a guiding framework for community engagement and the course development process (Figure 2). The CPAM is an evidence-based model that prioritizes collaborative adaptation to design culturally responsive and locally contextualized curricula [22,23]. The CPAM is a strong fit for Indigenous health because it emphasizes equitable collaboration and community-led cultural adaptation of interventions through a stepwise iterative process. The ability to align with community priorities made it well suited for a community-focused health literacy training program.

#### 2.1.1. Step 1: Establish Collaboration

The BMWIC program was ideated in direct response to the MH-related needs identified by the FBC. Its development was to address two priorities: (1) fulfill the FBC’s request and (2) contribute to knowledge generation through a culturally aligned, community-engaged educational program. The principal investigator (PI) and on-site program manager, both members of the FBC, were key liaisons in acquiring buy-in and approvals from key stakeholders and leaders, as well as determining collaborators based on their existing networks.

The FBC Public Health Nurses (PHNs) were identified as being most aware of community MH needs and workforce burnout. Therefore, they were invited to participate in several focus group discussions (FGs) as part of a larger Community of Practice (CoP) for the project. FGs were analyzed via a thematic analysis approach, whereby three coders synthesized core themes which formed the basis for the BMWIC program learning objectives [24].

Additionally, the Johns Hopkins Center for Indigenous Health (JHCIH), housed in the Bloomberg School of Public Health, was a collaborator throughout the development process, providing technical support on course development, administrative planning, processes for acquiring institutional course approvals, enrollment processes, learning platform options, and mode of delivery. After much thought and consideration across collaborators, it was decided the BMWIC program would be housed within the JHSON as it is a nurse-led initiative developed in partnership with the FBC PHNs.

#### 2.1.2. Step 2: Review Effective Educational Models

We began by assembling a multidisciplinary course development team (CDT) who oversaw Steps 2 through 5 in accordance with their role and area of expertise. The team comprised the project lead, course coordinator, curriculum development expert, two instructional designers, one evaluator, five external guest faculty, and five faculty from JHSON and JHCIH. Subject matter expertise included psychiatric nursing, public health, case management, social work, Indigenous health, AI law, and AI/AN culture. Indigenous faculty with expertise in cultural safety and Tribal health systems were central to culturally aligned content development, while psychiatric-mental health nursing faculty contributed to curriculum adaptation and learning design.

The JHSON Psychiatric Mental Health Nurse Practitioner (PMHNP) Certificate program curriculum served as a starting point, originally approved by the university in 2013 and is accredited by the Commission on Collegiate Nursing Education (CCNE) [25]. The program emphasizes socio-ecological contributions to stress and health and has an emphasis on integrated community MH. The JHSON utilizes a whole-person assessment framework that guides health care providers in identifying physical, behavioral, psychological and environmental contributions to symptoms and targets for interventions [26].

While the BMWIC program was not intended exclusively for nurses, we next reviewed standard nursing educational standards and competency-based benchmarks to establish competency domains (Table 1) that were used as guideposts to inform curriculum development and adaptation. These included the American Association of Colleges of Nursing-The Essentials: Core Competencies for Professional Nursing Education, the American Nurses Association-Psychiatric Mental Health Nursing Scope and Standards of practice, the National Organization of Nurse Practitioner Faculties, and the American Psychiatric Nursing Association Educational Crosswalk Toolkit [27,28,29,30]. Together, these standards helped ensure that the curriculum was organized, evidence-based, and aligned with core MH competencies while remaining applicable to a multidisciplinary audience.

The next step involved exploring similar MH education programs, of which two were identified. MH training resources designed for Indigenous communities are offered by the Indian Health Service (IHS) and Johns Hopkins Center for Indigenous Health (JHCIH). Free and accessible online training provided by IHS includes Mental Health First Aid; Cultural Safety; Question, Persuade, Refer (QPR); Trauma-Informed Care; and Applied Suicide Intervention Skill Training (ASIST) [31]. The JHCIH is another resource delivering training and education specific to the health needs of AI/AN communities, including a summer/winter institute course on harm reduction [32]. The JHCIH offers 14 institute courses and has trained over 4000 graduate students and 47 Indigenous staff/faculty to date. Public health training, including many of the ones reviewed, is often designed from large population- or systems-level perspectives and neglects to include local community knowledge and experience.

The Instructional Designers ensured that Universal Design for Learning principles were prioritized and incorporated throughout the curriculum. These principles support equitable access and success for all learners by addressing three main dimensions of learning: engagement (the why), representation (the what), and action and expression (the how) [33]. This aligns with culturally responsive pedagogy for AI/AN learners because it emphasizes flexibility, relevance to learners’ cultural and historical contexts, and the creation of safe, inclusive learning environments. It is also particularly well suited for working adult learners, who often engage with course content across devices and within competing personal and professional demands. 

The draft BMWIC course outlines and learning objectives were reviewed by the JHSON curriculum committee and the Non-Degree Non-Credit (NDNC) committee. The overarching competency domains provided the starting points for adapting and developing tailored content designed to address AI/AN community MH resource gaps and strengthen learner MH literacy (Table 1). Though the competency domains are reflective of nursing-specific competencies, the BMWIC course-specific objectives and activities have been further tailored to match learners’ role(s) and broader scopes of multidisciplinary practice within the community.

#### 2.1.3. Step 3: Assess Cultural Acceptability

The BMWIC program places greater emphasis on cultural congruity of content to community-identified needs than most Western, biomedical training programs. This required extensive community engagement and Indigenous health subject matter expertise throughout the curriculum development process. Because the AI/AN-specific cultural elements were not already present in the JHSON PMHNP curricula, these components had to be co-created with community partners. Additional distinctions included a more robust Tribal ethical review process, intentional development for a multidisciplinary learner audience, and the use of strength-based content that went beyond simple adaptation to reflect different ways of knowing and being.

Aaniiih and Nakoda culture and place-based considerations were included in the course design to address the training priorities and educational needs identified by the FBC PHNs in Step 1. The curriculum was purposefully designed to be culturally safe and aligned, with learning activities grounded in AI/AN strength, respect, rigor and relevance. For example, course content includes a clear overview of the impacts of colonization and historical trauma on present-day AI/AN MH disparities and inequities.

To assess the cultural acceptability of the program, local community-based healthcare leaders and cultural experts participated in reviewing the curriculum. These reviewers focused on the relevance of course content to community needs and priorities, accuracy, and cultural alignment (Table 2). Fidelity to the collaborative partnership was maintained through shared decision-making and responsibility for developing high-quality, culturally safe, and relevant curricula. Community reviewers also assessed and modified the content for appropriateness, accuracy, and cultural safety by carefully examining the language, concepts, and titles used in the learning objectives and course submodules.

#### 2.1.4. Step 4: Adapt Curriculum

The JHSON program was not originally designed for the cultural context or learning needs of FBC members, nurses, CHWs, and other healthcare workers. Accordingly, the CPAM process required extensive community consultation to adapt the curriculum to broaden comprehension across differing learner educational levels, including cultural priorities while centering local MH-resource-gap-related needs. Task-shifting principles were incorporated to support practical skill development and strengthen local care delivery. As a result, the adapted BMWIC differed from a graduate-level clinical course in both content and delivery, using a customized curriculum for a diverse learner audience.

Content for curriculum adaptation drew from four primary sources: relevant components of the JHSON PMHNP program, FGs with FBC PHNs, the evidence-based literature and findings from a mixed-methods study conducted in the FBC [9]. The iterative process prioritized community-identified needs, particularly closing the MH resource gap, improving MH literacy, and addressing high workload and caregiver burnout. To respond to burnout concerns, the team used a trauma-informed approach and integrated self-care strategies into learning activities to support caregiver wellness.

Course materials were designed to meet Web Content Accessibility Guidelines (WCAG) 2.0, Level AA standards. Accessibility features include human-generated closed captions, alternative text for images, clear heading structures for screen readers, sufficient color contrast, consistent navigation, and chunked content to reduce cognitive load. Plain language principles were applied throughout, with materials written at approximately an 8th-grade reading level to support learners with varying literacy and backgrounds.

Because the curriculum was intended for a rural audience with limited connectivity, it was also designed for low-bandwidth environments and mobile access. Strategies included short modular lectures, downloadable slides/handouts for offline use, and simplified course page design to reduce load times and scrolling burden. Additional features included clearly stated learning objectives, module introductions and summaries, and estimated completion times to support learner pacing and orientation.

Following completion of all curriculum adaptations, community MH experts and healthcare leaders from FBC then reviewed the curriculum in its entirety—four courses in total. This review process occurred over four months, from November 2024 to February 2025, and was facilitated by the JHSON CDT in collaboration with FBC representatives. Recommendations from the FBC reviewers were incorporated into the final course map to facilitate building out course-specific activities, which occurred between April 2025 and December 2025.

CDT faculty Indigenized course content while maintaining cultural integrity and academic rigor. This was done through culturally aligning learning activities and collaboration with Indigenous guest faculty. This process supported the respectful integration of IWK into lessons on spiritual, ceremonial, health, and wellness practices, with cultural alignment and safety reviewed by FBC collaborators. As an example, one learning activity exemplified an Indigenous wellness framework called the Sweetgrass Method, developed by Dr. Mark Standing Eagle Baez for Course 3: Psychiatric Care Settings and Interventions in American Indian Communities [34,35]. This method promotes introspection as a healthcare provider in terms of acknowledging biases and developing a plan for patient-centered communication and continuity of relationship to the treatment plan [35].

Another framework of health that is part of Aaniiih/Nakoda cosmology is the Medicine Wheel [36]. Describing AI health frameworks such as the Medicine Wheel aligns with the JHSON whole-person framework and provides an example of how the CDT faculty culturally adapted content. The Medicine Wheel philosophy is an example of curricular cultural adaptation because it grounds MH learning in an Indigenous framework of wellness. By connecting physical, mental, spiritual, and social well-being with the four cardinal directions, it offers learners a holistic way to understand MH concepts. This approach helps learners see MH not as an isolated issue, but as part of an interconnected system of healing that is culturally meaningful and relevant to AI/AN communities. This process of making content culturally relevant and meaningful strengthens trust and supports shared decision-making between the CDT and the community. These culturally derived models will bridge the sometimes foreign or stigmatized aspects of MH concepts for learners as they engage with the content.

#### 2.1.5. Step 5: Evaluation Plan

A comprehensive, multi-method evaluation plan was established to pilot the BMWIC program at a later time. A structured evaluation process with predefined indicators and systematic data collection was designed to measure changes in participant knowledge (primary outcome) and impact on academic goals and career application/advancement (Table 3). A change in knowledge measure will be administered to enrolled students before and after each course. The mean, median, and percent increase in scores will be assessed and shared with course faculty after each course to understand learners’ mastery or comprehension of course learning activities. Program acceptability, feasibility, and learning platform usability will be assessed using multiple instruments administered before and after each course, as well as at program completion. Cultural sensitivity of the curriculum, an especially important consideration for an AI/AN-serving population, will be assessed through surveys administered after each of the four courses. Cohort retention, defined as the percentage of learners who complete the program, will be used as an indicator of feasibility and effectiveness. In addition, key informant interviews or focus group discussions with learners will be conducted at the end of the program to further assess effectiveness, acceptability, feasibility, and usability. All data will be collected via surveys through Research Electronic Data Capture (REDCap), a secure web application for collecting and managing research data. Qualitative data will be collected during post-course interviews. Audio recordings will be professionally transcribed and data thematically analyzed using NVivo, a qualitative data management software.

Fidelity of the pilot implementation will be tracked by how closely learning objectives and newly developed curriculum follow the CPAM principles: participatory collaboration, cultural alignment, and the agreed upon curriculum development and adaptations. Community collaboration and involvement thus far have facilitated close alignment to CPAM principles; however, data collected from BMWIC learners will corroborate whether adaptations made to the curriculum, and the courses overall, are aligned with the community priorities and needs.

Due to workforce shortages, it is likely that in a rural, Tribal community pilot implementation, the enrollment may be 10 learners or less. Once there are sufficient students in a single cohort or multiple cohorts, statistical methods may be used to determine longitudinal change. Centrality measures (mean, median) for group achievement and percentage change for individual knowledge gain can be determined by student scores on assessment tasks centering course competencies. Additionally, learner demographics, collected at enrollment, can be used to descriptively understand cohort characteristics and experience in the program.

Implementation of this evaluation plan will provide valuable information for future refinement of the program and/or courses that are needed prior to considering scaling and adapting the program to additional populations in Tribal, urban, and/or IHS settings.

#### 2.1.6. Step 6: Pilot and Scale

The learner audience for this educational intervention is atypical and diverse and will include PHNs, CHWs, and healthcare leaders who work in the FBC or surrounding areas. They will be recruited via dissemination of flyers, email, and word of mouth invitations made—leveraging the CDT’s networks.

Each pilot participant will be enrolled in the program at no cost to them. They will obtain a JHSON email address and will be enrolled onto the online JHSON CANVAS learning management system by the course coordinator and the project lead. This will enable access to the university’s library resources and all course materials. The program will be run in sequential order over the course of one year, whereby each course will be completed in 10–12 weeks with a break between each one to facilitate self-care. Each learner who successfully completes all learning requirements in the four-course series will receive a Certificate of Completion and a monetary incentive of $200 as a token of appreciation for their evaluative feedback.

### 2.2. Ethical Considerations

The Aaniiih Nakoda community was engaged in every aspect of developing the BMWIC program. An extensive community engaged research process began with obtaining approvals from the Tribal Advisory Board (TAB), Johns Hopkins University Institutional Review Board (IRB), Aaniiih Nakoda College (ANC) IRB and from FBC Tribal Health program directors. The ANC IRB approves all research-related projects in the FBC to ensure alignment with cultural and data sovereignty protocols. All data related to this collaboration belongs to the FBC. Special requests or inquiries about this protocol, the BMWIC program and/or future pilot data require review and approval by the aforementioned parties.

## 3. Discussion

### 3.1. Program Significance

The FBC’s primary goal in partnering with the JHSON team was to enhance MH literacy and address contributors to the MH resource gap. Educational programs designed by the community to promote MH and wellbeing in AI/AN communities are available; however, few offer the breadth, relevance, and diversity of perspective found in the BMWIC program. The BMWIC program offers valuable contributions and insights for the development of educational programs designed for AI/AN communities, with implications for nursing, public health, and MH practice.

The socio-environmental infrastructure of rural communities remains a barrier to recruiting MH providers in MH shortage areas. Taken together, these findings highlight that the MH care gap in the FBC is not only a workforce issue, but a systems issue that requires solutions beyond traditional provider recruitment alone. In this context, task shifting offers a practical and culturally responsive way to extend care by supporting trusted community responders and existing frontline workers to take on selected MH screening and referral functions. AIAN-serving health systems, including Indian Health Service facilities, “638” autonomous Tribally operated health centers, and Urban Indian health centers, experience health provider shortages across all specialties [37]. Rurality of hospitals, clinics and 638 facilities poses several challenges for health providers who wish to care for AI/AN communities. Housing is often limited and difficult to obtain for the healthcare workforce. Living in remote locations often requires long commutes and offers limited entertainment, shopping, dining, and access to health care compared with urban areas.

The FBC is challenged by rurality and insufficient workforce training in MH specialty fields. Concentrated poverty is also a salient barrier to accessing care. Therefore, the BMWIC program is an actionable solution toward enhancing MH literacy and applying task-shifting strategies across the community to circumvent some of these challenges. This solution emphasizes how expansion of local knowledge and capacity offers a form of immediate local intervention and empowerment designed to address the MH crisis in AI/AN communities. Health-related education options are more successful when grounded in community assets, rather than relying solely on national public health initiatives. Knowledge itself is both a privilege and an intervention. Although training and educational resources designed for AI/AN communities exist, few programs truly reflect locally identified needs, priorities, and strategies for change.

Additionally, the BMWIC program offers a multidisciplinary training model that prioritizes cultural safety. Inter-disciplinary and cross-cultural collaboration promote opportunities for knowledge exchange while strengthening clinical, relational and culturally grounded knowledge and insights. An inclusive, multidisciplinary approach broadens and deepens what learners gain, enriches the learning experience, and produces more relevant, responsive and effective interventions for the communities served [38]. Cultural safety is essential for addressing anti-Indigenous racism and improving inequitable health and MH care experienced by Indigenous peoples. It concurrently supports improved patient care skills of allied providers by bridging understanding, values, and priorities between Indigenous patients and allopathic systems of care [16]. To create an inclusive and non-discriminatory learning environment, cultural safety was prioritized in both the curricular development and delivery of the BMWIC program.

### 3.2. Strengths of the Curriculum Design

Several key factors contribute to the overall strength in design of the BMWIC educational program. Strengths of this curriculum design that prioritize community needs and maximize learner outcomes include considerations for reading comprehension, accessibility, the digital divide, and cultural alignment. Selection of the CANVAS online learning platform supports the community’s needs for a flexible and accessible learning environment. The design was tailored to the needs of community-based learners who are often working full time and require a flexible, self-paced learning opportunity. The CPAM is an ideal fit for partnerships that are community-led.

The content in the BMWIC courses was designed to directly address the mental health crisis in AI/AN communities utilizing a community-led, strength-based and culturally aligned knowledge sharing and curriculum development approach.

When offered as a for-credit course, continuing medical/nursing education credit or as a certificate program, the community-level responders and healthcare workforce gain a highly specialized knowledge base and skill that may positively impact the MH of AI/AN communities. Greater insights on IWK and practices are valuable resources that will support AI/AN wellness and life-sustaining practices.

The BMWIC program has been developed with good intentions and integrates IWK and cultural wellness practices and this was only achieved through strong partnerships with Indigenous knowledge keepers and community partners. In addition, the Indigenous faculty leading the project worked diligently to maintain a high level of fidelity to creating a culturally rich and informed curriculum that would feel safe for AI/AN communities. The BMWIC integrates Indigenous health-related knowledge systems with existing Western MH screening and care coordination approaches. This integrative approach for designing an educational intervention increases access to opportunities for those interested in learning about practical and culturally aligned approaches for health and wellbeing in AI/AN communities.

This educational program directly counters systemic racism and the exclusion and marginalization of Indigenous knowledge systems. Traumatic and structural issues that are embedded in academic institutions have resulted in the rejection and restrictriction of IWK. The BMWIC program demonstrates an opportunity for intergenerational transfer of life-sustaining Indigenous ontologies of health that have been disrupted by colonization. Indigenous knowledge must remain protected and respected while also ensuring future generations have access to health sustaining knowledge. Making knowledge accessible and simultaneously protecting the origins of Indigenous knowledge is a responsibility of educators, healthcare providers and researchers. The BMWIC program targets a broader learner audience to maximize impact and promote health across AI/AN communities. The CDT prioritized protection of and respect for sacred Indigenous wisdom and all content was approved by community including the respectful inclusion of sacred discussions on Indigenous spiritual and ceremonial self-care interventions. As a response to community, this program makes knowledge accessible, while recognizing the physical and emotional vulnerability of front-line responders and the healthcare workforce who work in underserved Indigenous communities.

### 3.3. Limitations

Institutional and systemic barriers were among the greatest challenges in designing the BMWIC program. The curriculum development phases (Steps 2–4) were constrained by short timelines and systemic or procedural hurdles that proved time consuming, discouraging, and at times marginalizing. Funding deadlines and institutional processes placed time constraints on the CDT’s ability to develop the curriculum with the full rigor and scope intended. Bloom’s taxonomy was utilized to ensure measurable action verbs were incorporated in the learning objectives, but the constrained timeframe limited the teams capacity to fully operationalize learning activities that match each specific learning objectives. However, it is anticipated the pilot and evaluation feedback will provide guidance on those and other refinements needed ahead of the full launch of the program.

A key element that slowed the BMWIC program progression was having a limited number of staff involved from start to finish; much of the early program development rested on the project lead and a project assistant. Sufficient faculty and program support members were brought onto the CDT during early planning phases and during curricula development. For future refinement and iterations, it will be important to begin with a consistent, fully staffed implementation and faculty team to ensure program integrity and responsiveness. Additionally, having a full-time program coordinator will be important for managing day-to-day operations, learner recruitment/enrollment, and community partnerships. Engaging faculty who are knowledgeable of AI/AN health issues is essential to develop and deliver rich and relevant content, perform course updates, assess learning, and engage with and mentor the learners. Additional staff recommendations include: (1) a full-time program evaluator for monitoring fidelity to learning objectives and outcomes and conducting robust evaluations and (2) a robust administrative support staff for supporting registration, marketing, curriculum revisions, and acquiring additional funding.

Importantly, the curriculum’s feasibility and acceptability measures are not able to assess community-level impacts of the program. Future iterations should plan on evaluating and examining whether the program translates into measurable community wide MH-related impacts by incorporating clincial outcome measures like MH screenings, referrals, and utilization.

### 3.4. Impact

Institutional support from JHSON and the FBC has provided an opportunity to develop an educational intervention designed to improve MH outcomes through training and development of those working in AI/AN communities. Higher learning institutions, Tribal Behavioral Health programs, and the IHS are examples of organizations that may benefit from the use of this four-course curriculum. It can be implemented parallel to existing institutional efforts to enhance quality care and offer culturally informed and safe MH care delivery and prevention activities for AI/AN communities. However, the uses and implementation of this protocol may reach far beyond the FBC and MH spaces, as this protocol outlines a rich, informative, and community-derived process which may be adapted for any area/population experiencing significant workforce shortages and/or adverse outcomes. Feedback acquired in the development of the BMWIC program, and that still to come from the pilot, will be extremely valuable for ensuring the training provided—with the intention increasing MH knowledge and literacy, care coordination, and culturally responsive care delivery—is, in fact, meeting the need.

## 4. Conclusions

The current protocol serves as a roadmap for the development of the BMWIC program, which undertook an approach paralleling the objectives of the Indian Self-Determination and Education Assistance Act (Public Law 93-638). This legislation aims to create opportunities to enhance Tribal capacity for greater local control for the purpose of implementing quality and culturally aligned healthcare services [39]. The integration of IWK into a practical training program directly opposes colonial agendas that were intended for intentional destruction or suppression of Indigenous knowledge systems. This approach to intervention curriculum design also utilizes a World Health Organization (WHO) strategy to leverage Traditional Medicine and local cultural knowledge to fill healthcare gaps in provider shortage areas [40]. This work is grounded in the perspective of nursing—consistently ranked as the most trusted profession—and further strengthened by the leadership of Indigenous nurse scientists, whose advocacy and lived experience advance culturally relevant care for their communities.

Ultimately, the long-term goal of the BMWIC program is to reduce the MH resource gap in AI/AN communities and may contribute to reduced psychiatric-related morbidity and mortality and support more sustainable, community-based MH care for underserved Tribal populations. Future iterations and adjacently developed programs may be scaled to other healthcare, academic and community-based settings. This work has the capacity to benefit interdisciplinary healthcare professionals and scientists by providing valuable knowledge and skills to address limitations in health system infrastructure. The knowledge gained from these courses is critical to maximize cultural safety in clinical practice and health science education, and develop inclusive and transformational policy that supports self-determination for Indigenous people and promotes health equity for all.

## Figures and Tables

**Table 1:** Alignment of BMWIC competency domains, community needs, and courses.

BMWIC Competency Domain	Community Need(s)	Course 1	Course 2	Course 3	Course 4
Cultural Safety, Historical Trauma, and Health Equity	Mental health literacy; culturally informed care; historical trauma; systemic discrimination	✓	✓		✓
Lifespan Mental Health and Neurobiology	Mental health literacy; prevention across the lifespan	✓	✓		
Evidence-Based Psychopharmacology	Access to culturally informed mental health care	✓		✓	
Therapeutic Communication and Care Coordination	Care coordination; access to care; self-determination				✓
Evidence-Based Practice and Health Technology	Capacity building; workforce development	✓		✓	
Psychiatric Assessment, Diagnosis, and Clinical Decision-Making	Targeted training; provider shortages; task shifting		✓	✓	
Advocacy, Leadership, and Social Justice	Systemic discrimination; self-determination; poverty	✓			✓
Systems of Care and Community-Based Psychiatric Practice	Rurality; community health infrastructure; access to care		✓	✓	✓
Documentation, Quality Improvement, and Interprofessional Collaboration	Care coordination; capacity building		✓	✓	✓

Note. Competency domains represent adapted PMHNP curriculum domains and are presented in summarized form to preserve institution-specific learning objectives while illustrating curriculum alignment.

**Table 2:** Alignment of FBC priorities with BMWIC courses by socioecological determinants of MH.

Community-Identified Priorities and Socioecological Determinants of Health	BMWIC Courses			
	Course 1	Course 2	Course 3	Course 4
Education				
MH Literacy	✓			
Targeted Training	✓	✓	✓	
Mental Health First Aid			✓	
Economic				
Community Health Infrastructure		✓		
Capacity Building	✓	✓	✓	✓
Poverty				✓
Geographic				
Rurality/Remote Issues				✓
Health Provider Shortage		✓		
Access to Care				
Culturally informed MH care	✓			
Task Shifting		✓	✓	
Care Coordination		✓		
Traumatic and Structural Issues				
Youth suicide		✓	✓	✓
Intergenerational/Historical Trauma		✓		
Systemic Discrimination and Racism	✓			✓
Self Determination		✓		✓

**Table 3:** Evaluation measures for BMWIC program by timepoint.

Measure(s)	Type of Data Collection	Timepoints			
		Pre-Program	Course Start	Course Complete	Post-Program
Student Demographics					
Gender identity Tribal affiliation Professional position Level of education Skills desired from BMWIC program Anticipated impact of BMWIC program on job or academic performance	Quantitative: Survey items (Categorical variables) Qualitative: Open-ended response survey items	✓			✓
Knowledge Growth					
Course competencies	Quantitative: Survey items (% correct, pre/post)		✓	✓	
Learning Environment					
In-person vs. virtual, synchronous vs. asynchronous learning environment Course length and format Program length and format Level of support from assigned mentor Time spent weekly on course content	Quantitative: Survey items (Categorical variables) Qualitative: Open-ended response survey			✓	
Cultural Integration					
Culturally Sensitive Curricula Scale-Revised (CSCS)	Quantitative: Survey battery (Sum score)			✓	
Work/Life Integration					
Barriers and facilitators to program completion	Qualitative: In-depth interview or FGs				✓
Feasibility, Acceptability, and Usability					
Feasibility of Intervention Measure (FIM) (4-items) Acceptability of an Intervention Measure (AIM) (4-items) System Usability Scale (SUS) (10-items)	Quantitative: Survey batteries (Sum scores)			✓	
Reach and Personnel					
Number of students matriculated into BMWIC program pilot BMWIC program completion rate Number of BMWIC faculty Number of Indigenous faculty Number of mentors engaged	Quantitative: Continuous variables	✓		✓	

## Data Availability

No data is available because this is a protocol paper that outlines the development, pilot implementation, and evaluation of the BMHWIC program. Any inquiries about this protocol or the educational program must be directed to the principal investigator and the Aaniiih Nakoda Institutional Review Board for review and approval.

## Public Involvement Statement

Extensive involvement of the FBC was an important part of the process. Once the FBC, along with Principal Investigator Dr. Teresa Brockie, established the need for the BMWIC program, the process involved engagement and consultation at various levels. Through community-engaged research, the idea to train PHNs and CHWs in MH literacy was established, and subsequently priorities for curriculum components and prospective student needs.

## Guidelines and Standards Statement

This manuscript was not drafted using a protocol reporting guideline.

## Abbreviations

The following abbreviations are used in this manuscript:

BMWIC	Behavioral/Mental Health and Wellbeing in Indigenous Communities program
MH	Mental Health
FBC	Fort Belknap Community
JHSON	Johns Hopkins School of Nursing
PMH	Psychiatric Mental Health
IRB	Institutional Review Board
FBC-JHSON	Fort Belknap Community-Johns Hopkins School of Nursing
AI/AN	American Indian and Alaska Native
HPSA	Health Provider Shortage Area
IHS	Indian Health Services
CHW	Community health worker
RN	Registered Nurse
CoP	Community of Practice
PMHNP	Psychiatric Mental Health Nurse Practitioner
NARCH	Native American Research Centers for Health
CPAM	Collaborative Participation Adaptation Model
ANC	Aaniiih Nakoda College
TAB	Tribal Advisory Board
FGs	Focus groups
NDNC	Non-Degree Non-Credit
CDT	Course Development Team
JHCIH	Johns Hopkins Center for Indigenous Health
PHNs	Public Health Nurses
DSM	Diagnostic and Statistical Manual of Mental Disorders
ICD	International Classification of Disease
NONPF	National Organization of Nurse Practitioner Faculties
APNA	American Psychiatric Nursing Associations
UDL	Universal design for learning
PL	Plain language
WCAG	Web Content Accessibility Guidelines
CSCS	Cultural Sensitivity curriculum scale
FIM	Feasibility of Intervention Measure
AIM	Acceptability of Intervention Measure
REDCap	Research Electronic Data Capture
NIH	National Institutes of Health
